# Social-political and vaccine related determinants of COVID-19 vaccine hesitancy in Tanzania: A qualitative inquiry

**DOI:** 10.1371/journal.pgph.0002010

**Published:** 2023-06-14

**Authors:** Sally Mtenga, Grace Mhalu, Brianna Osetinsky, Kaushik Ramaiya, Tani Kassim, Brady Hooley, Fabrizio Tediosi

**Affiliations:** 1 Ifakara Health Institute, Dar es Salaam, Tanzania; 2 Swiss Tropical and Public Health Institute (Swiss TPH), Allschwil, Switzerland; 3 University of Basel, Basel, Switzerland; 4 Shree Hindu Mandal–Hospital, Dar es Salaam, Tanzania; Institute of Development Studies, UNITED KINGDOM

## Abstract

Vaccines have played a critical role in the response to the COVID-19 pandemic globally, and Tanzania has made significant efforts to make them available to the public in addition to sensitizing them on its benefit. However, vaccine hesitancy remains a concern. It may prevent optimal uptake of this promising tool in many communities. This study aims to explore opinions and perceptions on vaccine hesitancy to better understand local attitudes towards vaccine hesitancy in both rural and urban Tanzania. The study employed cross-sectional semi-structured interviews with 42 participants. The data were collected in October 2021. Men and women aged between 18 and 70 years were purposefully sampled from Dar es Salaam and Tabora regions. Thematic content analysis was used to categorize data inductively and deductively. We found that COVID-19 vaccine hesitancy exists and is shaped by multiple socio-political and vaccine related factors. **Vaccine related factors** included worries over vaccine safety (e.g., death, infertility, and zombie), limited knowledge about the vaccines and fear of the vaccine’s impact on pre-existing conditions. Participants also found it paradoxical that mask and hygiene mandates are expected even after vaccination, which further exacerbated their doubts about vaccine efficacy and their hesitancy. Participants possessed a range of questions regarding COVID-19 vaccines that they wanted answered by the government. **Social factors** included preference for traditional and home remedies and influence from others. **Political factors** included inconsistent messages on COVID-19 from the community and political leaders; and doubts about the existence of COVID-19 and the vaccine. Our findings suggest that the COVID-19 vaccine is beyond a medical intervention, it carries with it a variety of expectations and myths that need to be addressed in order to build trust and acceptance within communities. Health promotion messages need to respond to heterogeneous questions, misinformation, doubts, and concerns over safety issues. An understanding of country-specific perspectives toward COVID-19 vaccines can greatly inform the development of localized strategies for meaningful uptake in Tanzania.

## Introduction

COVID-19 remains an important health concern globally that requires a sustainable effort to reverse its negative impact [1–3]. Luckily, vaccines of proven efficacy have been developed [4–7]. By 31^st^ December 2020, the World Health Organization (WHO) had approved the first emergency use of the vaccine and allowed its use for the public [8]. So far, vaccines for COVID-19 have been proven to both prevent transmission of the infection and reduce the likelihood of developing severe disease and death, and are a main component of public health strategies to end the pandemic [9]. The WHO put forward a clear statement that ‘equitable access to safe and effective vaccines is critical to ending the COVID-19 pandemic,’ and encouraged global initiatives to support vaccine development in this emergency context [10]. While most high-income countries (HIC) have managed to immunize a large proportion of their populations, many low-income countries (LIC) have struggled to do so [11].

Tanzania reported the first case of COVID-19 in February 2020. Although the country did not implement lockdown measures, other preventive measures were implemented. Such measures included wearing masks, sanitizing hands and adaptation of complementary traditional remedies that were thought to boost immunity [12].

In Tanzania, COVID-19 vaccines could not be quickly implemented since the country did not have full capacity to deploy the vaccines. In addition, the former late President John Pombe Magufuli also insisted that the Ministry of Health need to conduct a robust evaluation before accepting the use of vaccines in the country [12]. However, efforts to prevent COVID-19 transmission were intensified under the leadership of Tanzania’s new president, Her Excellency Samia Suluhu Hassan, who came into power in May 2021. The Ministry of Health has advocated for COVID-19 vaccination and set the target to vaccinate at least 70 percent of its 60-million population by December, 2022 [13].

In July 2021, Tanzania received their first batch of COVID-19 vaccines, consisting of 1,058,400 doses of Johnson & Johnson (Jansen) vaccines donated by the US government and delivered through the COVID-19 Vaccines Global Access (COVAX) arrangement. The first batch of vaccine started to be distributed in July 2021 and by September 2021, the vaccines were available in 1,548 health facilities in Tanzania. Although vaccines have become widely available in Tanzania, a substantial number of Tanzanians are still undecided about getting vaccinated. Anecdotal data [14, 15] and reviews [16] show that COVID-19 scepticism and vaccine hesitancy exist in the country and can affect wider vaccination coverage.

Evidence suggest that community uptake of public health recommended interventions is not a smooth process [17]. This is due to various factors including community acceptance, inadequate prior information to inform the policy makers on what elements need to be considered before the implementation of the intervention [18, 19], and political environment may also be an important determinant of public acceptance of health intervention [20, 21] or successful implementation of an intervention [22].

For instance, in some high income countries, while there has been vaccine hesitancy, COVID-19 vaccine resistance appears to be influenced by deep political underpinnings [23, 24] with groups of people choosing not to be vaccinated [25, 26]. In 2021, the World Health Organization and the United Nations Children’s Fund (UNICEF) sounded the alarm based on the new data showing that global vaccination coverage continued to decline in 2021 [27].

In fact, similar hesitancy has been seen with the acceptance of Human Immunodeficiency Virus (HIV) testing and Anti Retrovirus Treatment (RVT) services in Tanzania and elsewhere [28]. What has been learned is that implementation problems can be country, culture, class, or even gender, specific and that it is important to understand considerations for acceptance or rejection of preventive measures by context including the different groups [29].

Vaccine hesitancy is the result of several intertwined factors. Recent evidence in Africa [30] found that misinformation and politicization of the COVID-19 pandemic can contribute to public hesitancy or mistrust of vaccines. Another study conducted in Thailand found that low education, lack of confidence in the healthcare system’s ability to treat patients with COVID-19, and a low number of new COVID-19 cases per day contribute to COVID-19 hesitancy [31].

Tanzania may receive more funding to support vaccine roll-out [32]. However, the support will not be useful if people remain hesitant about COVID-19 vaccines. Scaling-up the vaccination campaign requires more doses but also issues of vaccine hesitancy need to be addressed.

As of August 2022, in Tanzania, over 14.8 million people have been vaccinated constituting 24.7% of the population against a global average of 62.9% of the population [33]. Vaccination rates are on the rise globally, but current literature suggest that skepticism persists in various countries including in Tanzania [16, 34]. Therefore, to increase COVID-19 vaccination coverage in Tanzania, it is important to understand the local patterns of COVID-19 vaccine hesitancy. One way to do this is to generate localized evidence on people’s perceptions and views on the COVID-19 vaccine in Tanzania. Therefore, this study aims to explore the context-specific factors behind COVID-19 vaccine hesitancy in Tanzania.

## Methods

### Ethics statement

The study was approved by the institutional review board of the Ifakara Health Institute (reference number: IHI/IRB/No: 35–2020) and the National Institute for Medical Research of Tanzania (reference number: NIMR/HQ/R.8a/Vol. IX/3518). Written informed consent was obtained from all participants prior to taking part in the research.

### Study design

A cross sectional qualitative descriptive design was employed through face-to-face semi-structured interviews to allow an in-depth exploration of participants’ views, attitudes, perceptions, and their questions regarding COVID-19 vaccines. Semi-structured interview was an appropriate method to discuss sensitive subjects whilst allowing a degree of flexibility to investigate emergent issues that seem important to each participant [35].

### Study settings

The study was conducted in Dar es Salaam and Tabora regions in Tanzania. Dar es Salaam, a region located on the Eastern coast of Tanzania, is the main commercial city in the country and has better resourced health facilities and access to comprehensive quality care. Tabora is a region located at the central zone of Tanzania, which forms a semi-urban locality well served with the health facilities although not to the same standard as Dar Es Salaam. The choice of the study sites was motivated by the observations that the urban areas have been the epi-centre for COVID-19 [36–38]. Additionally, the study team’s existing collaborations with the chosen areas make these an ideal choice for the study due to the coordination of recruitment strategies. Differences between the levels of social services and health care in the two regions was expected to enhance variation of views regarding COVID-19 vaccines from people of diverse social-economic backgrounds.

### Study participants and sampling

The study participants were purposively selected to include adult men and women aged between 18 and 70 years old. The target population was reached through community leaders in each study community and based on their availability and willingness to participate in the study. We interviewed participants until when we felt that there were no new information emerging during the interviews [39]. Finally, we were able to interview twenty-seven participants in the semi-urban context and twenty-nine participants in the urban context. Most participants in the urban area were of age 40–49 followed by those of age 20–29. Most participants in the semi-urban area were of age 30–49 followed by those of age 20–29. Participants of secondary level education were many in urban setting (38%). More details about participants’ social demographic characteristics are found in Table 1 below.

**Table 1:** Social demographic characteristics of the respondents.

**Urban**		**Frequency**	**Percentage**
	**Gender**		
	Males	14	48%
	Female	15	52%
	**Age**		
	20–29	8	28%
	30–39	7	24%
	40–49	10	34%
	50–59	4	14%
	**Marital Status**		
	Married	23	79%
	Unmarried	6	21%
	**Education**		
	Primary	8	28%
	Secondary level	11	38%
	Diploma/University	2	6%
	Unknown	8	28%
	**Occupation**		
	Skilled labor	21	73%
	Unskilled labor	6	20%
	Unknown	2	7%
Semi-urban		Frequency	Percentage
	**Gender**		
	Males	9	33%
	Female	18	67%
	**Age**		
	20–29	7	8%
	30–40	13	49%
	40–49	6	20%
	50–59	3	32%
	70+	1	1%
	**Marital status**		
	Not Married	4	15%
	Married	14	52%
	Separated/ widows	6	22%
	N/A	3	11%
	**Education**		
	Primary	19	70%
	Secondary level	4	15%
	Diploma/University	1	4%
	Unknown	3	11%
	**Occupation**		
	Skilled labor	4	14%
	Unskilled labor	1	4%
	Unknown	22	82%

### Recruitment

Local leaders, including the district officials in the respective study sites, were requested to assist in the recruitment of eligible participants. Prior to recruitment, the research assistants approached community leaders to inform them of the purpose of the study to ensure objectivity in the recruitment process. Local leaders were asked to request community members to volunteer to participate in the study by explaining to them about the importance of their participation and the purpose of the study. Leaders asked interested potential participants to provide their phone numbers and time at which they could be reached. Using this initial list, with contact information generated by community leaders, the research assistants contacted potential participants to formally ask if they consented to join the study. The decision to participate in the study was voluntary and a written consent was completed.

### Data collection

The data collection was implemented in October 2021. Face-to-face interviews were conducted using a semi structured interview guide to explore views and perceptions around COVID-19 vaccines. Experienced research assistants conducted the interviews after being oriented to the study objectives, data collection tools, and on the basics of research ethics. Senior social scientists (first and second authors) strictly supervised all data collection processes. The interviews took between 45 minutes and 1hour. Participants were interviewed at places of their choice where they felt there was adequate privacy. Most participants preferred to be interviewed in their homes. The interviews were recorded with the participant’s consent. The interviews took place in strict adherence to COVID-19 preventative measures such as wearing masks and using sanitizers. All the interviews were conducted in Kiswahili-a language that is mostly spoken and understood in Tanzania.

### Data analysis

All recorded qualitative data were transcribed verbatim in Kiswahili since the analysts were Tanzanians and well conversant with the language. Transcripts were frequently reviewed by 2 researchers to identify key themes that emerged from the data. Thematic Content Analysis [40] was conducted to identify inductive themes related to participants responses on the factors influencing their vaccine hesitancy. This process involved collapsing codes to fall under identified categories that portrayed the similar patterns of meaning. Then, the inductive themes were coded into broader theoretical categories such as ‘vaccine related factors’ and ‘socio-political factors’ and were adapted by generating sub-categories (Table 2) for a meaningful presentation of the findings on participant’s views regarding factors that influence COVID-19 vaccine hesitancy. Theoretical category such as social-political codes were adapted from the World Health Organisation (WHO) social-determinants of health framework which shows that the health status of people is an outcome of a wider social-economic and policy and political environments [41]. Common participant questions relating to COVID-19 vaccines were drawn from the data inductively and are presented in Table 2.

**Table 2:** Summary themes representing the views of participants relating to vaccine hesitancy.

Broader theoretical themes	Sub-themes
Social factors	◼ Preference for traditional and home remedies Influence from others
Political factors	◼ Inconsistent messages on COVID-19 from the community and political leaders Doubts about the existence of COVID-19 and vaccine due to inconsistent messages on COVID-19
Vaccine related factors	◼ Worries over vaccine safety (e.g., death, infertility, and zombie) ◼ Limited knowledge about the vaccines ◼ Fear of the vaccines’ impact on pre-existing conditions The need to maintain infection prevention and control (IPC) measures after COVID-19 vaccination ◼ Multiple questions regarding COVID -19 vaccines requiring answers by the government

To ensure reliability of the data, two steps were taken. Firstly, transcribed data were broadly reviewed by four experienced research assistants and two social scientists for familiarization and the identification of patterns and relevant themes corresponding to the topic guide. Secondly, two independent social scientists coded the same data to assess and establish inter-coder agreement. While coding the data, the social scientists cross-checked their coded data against the themes of the research assistants and another social scientist. Consensus on final themes categorization was reached in consultation with both social scientists.

Participant quotes relating to vaccine hesitancy were translated into English to support the presentation of the data.

## Results

We present the participants’ views categorized as socio-political and vaccine related factors influencing vaccine hesitancy in Tanzania. We also present the questions (Table 3) about the COVID-19 vaccines participants though the Government should address. We noted convergence of views regarding the vaccines from rural and urban areas.

**Table 3:** Participant’s common questions regarding the COVID-19 vaccine.

Number	Questions
1	Why should someone contract COVID-19 even after being vaccinated?
2	Apart from preventing COVID-19, what is the other diseases that can be prevented by the COVID-19 vaccine?
3	After receiving the first COVID -19 vaccine, how long should I stay to receive another vaccine?
4	Why people are not informed about the health conditions that makes them ineligible for COVID-19 vaccine?
5	Is there monitoring of side effects for those people who have been vaccinated? If yes, will the government make it open?
6	Why people are not insured in case they encounter severe risks from the COVID-19 vaccine?
7	Why the government is saying that it is not responsible for any outcomes from the COVID-19 vaccines?
8	Why COVID-19 vaccine has been developed fast as compared to other previous vaccines for other diseases?
9	Is the quality of the COVID-19 vaccines delivered to Tanzania similar to those delivered to other European countries?
10	Are there any long-term effects of the COVID-19 vaccines?
11	What are the main benefits that of the COVID-19 vaccines?
12	What does the COVID-19 vaccine protect against?
13	Is it true that if you are being vaccinated you can become infertile and unable to have children?
14	Can people with diabetes, people who take medication daily, or people with high blood pressure be vaccinated?
15	Can the COVID-19 vaccine be given to pregnant women?
16	Why can’t we have one type of COVID-19 vaccine? Why multiple vaccines?
17	Why should we not receive COVID-19 vaccine in the form of tablets instead of injection?
18	Why are we supposed to wear masks and use sanitizers after we have been vaccinated? Why should I live like a person who has never been vaccinated?
19	After being vaccinated, does it mean that you will not contract COVID-19?
20	Why has the country (Tanzania) been slow to accept the COVID-19 vaccine?

### Participant’s views on COVID-19 vaccine

#### Vaccine related factors

*Worries about vaccine safety*. In this study, concern about vaccine safety was reported to have contributed to vaccine hesitancy. Participants declared that their views on the acceptability of the COVID-19 vaccine was hindered by stories about the negative consequences of the COVID-19 vaccine. The most common negative consequences of the COVID-19 vaccine were related to death, infertility, blood clot, and becoming a “*ZOMBIE” (when someone change from being a normal human being to someone abnormal especially in their brain and body*. *Mostly like those people who died but believed to be alive and cannot perform things as normal human being*):

“*if you are vaccinated you can easily die”* [Male participant_semi urban].

*“if you are vaccinated you can experience blood clot”* [Male participant_Urban].

*“if you are vaccinated you can be infertile”* [Female participant_Rural].

*“if you are vaccinated*, *in the long run you can turn out to be ZOMBIE*” [Female participant_semi urban].

One participant reflecting on their concerns with the COVID-19 vaccines contended that the vaccines should be examined to assess their safety. The concerns about vaccine safety were based on ongoing stories about the negative consequences of vaccines as noted by the following participant:

*“These vaccines should be examined to see how safe they are in people’s health*. *This is because you hear people saying that today one can be vaccinated and tomorrow this person is gone (dead)*. *I have seen that people refuses vaccine because of people’s words (the recurred Swahili word was*: *‘Maneno maneno ya watu’)*, *you can also hear people saying that if you are vaccinated you can experience blood clot*, *so if such words spread in the streets people will not agree to be vaccinated* [Female Participant_Urban]”

Another participant noted that she was uncertain about the reality of such discourses although she still recommended that people should stick to Tanzanian herbs to prevent COVID-19:

“*We are not sure whether these news are true or not*, *you see*! *So it is better to use our own Tanzanian herbs to protect people from COVID-19*, *so that you do not see people today are okay and then tomorrow they are not there (dead)”* [Female participant_semi urban].

COVID-19 vaccine safety was questioned when the government announced that it would not be responsible for any harm caused by COVID-19 vaccines. To some people, this was an indication that vaccines were not safe, thus creating vaccine hesitancy:

*“I have heard the government saying that it will not be responsible for any consequences that will results from the vaccine*. *People who went for vaccination told us that even at the health facility the medical form that is filled by the nurses indicated that the government is not responsible of the vaccine side effects*. *This makes me ask several questions*: *why has the government refused to be held accountable for the consequences of the vaccine*, *if the vaccine was safe or in case the person experiences bad side effects (from the COVID-19 vaccine)*” [Male participant _semi-urban].

On the other hand, some participants were still hesitant, but they felt that the vaccine was safe because they had observed some people who had been vaccinated and had only mild symptoms. Others felt that the vaccine was safe because the newly elected president of Tanzania and other leaders were publicly seen receiving the COVID-19 vaccine, as noted by one female participant:

*“what make me see that this vaccine (COVID-19) is safe is because I have seen several leaders including our president Samia Suluhu going for vaccination*. *The leaders would not agree to tell the citizens to accept the vaccine which is unsafe but it is only that I have not made my decision to accept the vaccine*” [Female participant_Urban].

*Fear of vaccine’s impact on pre-existing condition*. Having pre-existing conditions was cited as one of the factors that made participants hesitant in accepting COVID-19 vaccine. Participants described not having enough information on the eligibility of people with pre-existing conditions to make informed decisions on whether to get vaccinated or not. One participant from the urban area made a specific request that clarification needs to be provided on whether people with chronic illness such as hypertension or diabetes are also eligible of COVID-19 vaccine:

*“I think these things need to be clarified*, *they should clarify to us on whether if someone has pressure [hypertension/high blood pressure]*, *diabetes or taking medicine for a long time*, *can also be vaccinated*. *We need to know whether if we are vaccinated while possessing such conditions*, *we will get problems*. *This is what makes us wait (not accepting vaccine)*. *This will open our minds and we will go for vaccine (COVID-19 vaccine)* [Female participant_Urban].

*The need to maintain infection prevention and control (IPC) measures after COVID-19 vaccination*. The need to continue instituting the IPC measures after vaccination cast doubts about the protective effect of the vaccine and this further contributed to vaccine hesitancy. Participants noted that healthcare providers also did not provide them with satisfactory responses as to why they need to continue wearing a mask after they had been vaccinated. They also described that a good vaccine would be one that would not necessitate the continuation of the IPC measures. This is demonstrated by the views of the following participant who expressed that the need for mask wearing after vaccination increased his doubt about the vaccine’s validity:

*“from my understanding*, *when someone is vaccinated need to be free from wearing masks*, *if you tell me to wear masks after I have been vaccinated this makes me doubt if that vaccine is valid*. *Why should someone who is vaccinated continue to wear masks or wash hands if the vaccine is protective*? *You already have vaccine in your body*, *why should you still wear masks*? *Why should I live like a person who has not been vaccinated*?*”* [Male participant_Urban].

In line with the above sentiment, another participant expressed his dissatisfaction with the responses provided by the health care providers regarding wearing masks after receipt of vaccination:

*“I am yet to be satisfied with the responses given by these health professionals*. *We have been asking why should we wear masks after receiving COVID-19 vaccines*? *the explanations given are not satisfying*, *they just say that the vaccinated people are preventing others from getting COVID-19 or if you wear masks you also prevent other people from getting COVID-19*. *Clarification on why after COVID-19 vaccination people should continue to use masks needs to be well elaborated because some of us expected that after being vaccinated it is direct [obvious] that you will not need to wear masks or sanitize your hands every time*. *They should bring a vaccine which will make you free after receiving vaccination*” [Female participant_Urban].

*Limited knowledge about COVID-19 vaccine*. Participants felt that their limited knowledge on COVID-19 vaccines influenced their vaccine hesitancy. They also reported that there is a need for people to understand the vaccine safety profile and the importance of the vaccine in reducing transmission and severity of disease, prior to accepting the vaccine. Reflecting on her vaccine hesitancy, one participant noted she needed more information before getting the vaccine while another participant noted the need to educate people more on the safety of the vaccines to increase the vaccine acceptance:

“*My interest before I accept the vaccine (COVID-19 vaccine) was first to know that if I am vaccinated*, *what will I be preventing*? *and how long will the vaccine stay in my body*?*”* [Female participant_semi-urban].

“*I speak the truth*, *education is still needed to help people understand the safety of the vaccine since most of us are still unclear about many stories that are ongoing regarding vaccine safety*. *That is why we are not accepting the vaccine* [Female participant_Urban].

#### Social factors

*Preference for traditional and home remedies*. Participants reported that the promotion of traditional medicine as an important strategy for prevention of COVID-19 also contributed to vaccine hesitancy. They mentioned that the government initially instructed people to use traditional herbs for the prevention and treatment of COVID-19. Some participants noted that they preferred to continue using their ginger steam as they believed it was sufficient to prevent the disease and thus did not accept the COVID-19 vaccine. Specific traditional remedies for COVID-19 prevention mentioned by participants included steam inhalation, traditional herbs, physical activity, sweet potatoes, lemons and ginger. One participant insisted he was using traditional medicine because he believed it was safer following guidance introduced at the onset of the pandemic in the country:

*“Just like the way I told you that we are using traditional herbs to prevent COVID-19*. *The COVID-19 vaccine was not there when the COVID-19 began*, *and our leaders told us that we need to practice steam inhalation*, *physical exercise*, *and natural food such as sweet potatoes*, *lemons and ginger (*..*)*, *and you know that these things are safer*, *but we are not sure about vaccines”* [Male participant_semi-urban].

*Influence from others*. A lack of acceptance of the COVID-19 vaccine and negative beliefs held by several people in the community appeared to influence vaccine hesitancy in others. This was noted by one male participant:

*“you know we human beings live by following what others are doing*. *We live by depending on others’ actions*. *So*, *if you see people are opposing this vaccine*, *you are also likely not to accept the vaccine*, *it is like you find yourself falling in the same hall*. *As of now*, *I do not see most young people accepting the vaccine*, *this also makes me hesitant of the vaccine (COVID-19 vaccine)*, *they say that you can only die of covid-19 if you are 50 and above”* [Male participant_Urban].

#### Political factors

*Inconsistent messages on COVID-19 from community and political leaders*. Participants reported contradictory messages coming from government leaders concerning the COVID-19 vaccine and this deterred many from receiving it. This inconsistency in messaging lead to increased doubts and hesitancy among study participants towards the COVID-19 vaccine. Participants felt that government officials needed to explain why there was a change in stance with regards to COVID-19 in order to encourage people to accept the vaccine. Even though many of the study participants reported inconsistency in COVID-19 messaging, a few others reported the political issues affected vaccine uptake. One participant explained his concern regarding the change in stance by government leaders, who initially appeared to discredit the vaccines and later started promoting them. This behaviour created some confusion on which position one should take regarding COVID-19 vaccine:

*“The same leaders who said the COVID-19 is bad in the past are the same who are now telling us that we need to be vaccinated*. *So*, *personally I fail to understand that which statement is true and we fail to understand where we should stand and what to take and what we should not take*. *In the past we were told that these vaccines are brought by white people and they (vaccines) are bad*, *but now this story has changed*, *we are now told to accept the vaccines*. *We are not sure whether we were deceived in the past or we were told the truth regarding the COVID-19 vaccine*. *To be honest personally*, *this bothers me so much*, *I am really stressed and I cannot make a decision on whether I should go for vaccination or not”* [Male participant_semi-urban].

A similar sentiment was provided by another participant who thought that it would be difficult to sensitize people to accept the vaccine due to inconsistent messages regarding COVID-19 vaccine by the leaders:

“*so how are we going to sensitize people to accept vaccine*? *Most of these people will tell you that last year leaders told us that we should not accept the vaccine*, *we should just do the inhalation*, *and people really hated the vaccine*, *then just after some months*, *you are now telling us to accept the vaccine*. *There is no big issue here*, *we have been vaccinated for years*, *but the issue now is when you see leaders comes up with different messages*. *I think the government should clarify that ‘initially we told you this’ and now ‘we are telling you this*, *because of this and that’*. *I know these people*, *want to understand first before they make a decision”* [Male participant_semi-urban].

Another participant had also expressed that the contradictory statements from the leaders that may hinder vaccine uptake:

*“Personally*, *I think this vaccine (COVID-19 vaccine) is okay*, *only that politics distort the good intension on vaccine*. *Vaccinations started long time ago*, *it is not today that we have started to receive the vaccines*. *Political leaders make people think that COVID-19 and all about vaccines is about business*, *because we keep on changing the conversation*, *today we say this*, *tomorrow we say that*. *The government should avoid politics on people’s health so that people are not confused”* [Female Participant_Urban].

*Doubts on the existence of COVID-19*. Participants’ narratives reflected doubts about the existence of COVID-19. Vaccine hesitancy was linked to the notion that COVID-19 has been created to fuel the businesses of aristocrats, and that the vaccines have nothing to do with the disease but are instead meant to spread COVID-19 and destroy the country’s economy. Some participants also cited a government commissioned investigation, when papaya tested positive for COVID-19, as evidence for their doubts about the disease’s existence. Speaking about hesitations on COVID-19 vaccines, one participant described the papaya story as follows:

*“in the previous government last year (2020)*, *the president had already announced that papaya was tested and found to have contracted COVID-19*. *What is still lingering in my mind is that how did COVID-19 enter into papaya*? *You see*, *the previous president told us to be watchful of the help we get from outside*, *and these COVID-19 vaccines are an example*. *See*, *there are two things here*, *one is that perhaps those COVID-19 vaccines are used to spread COVID in order to kill our economy*. *That is why it becomes difficult for us to accept those vaccines* [Male participant_semi-urban].

Another participant noted that her doubts on the existence of the disease, and subsequent vaccine hesitancy, was based on what she had heard in the community:

“*Some people that I know say that there is no COVID-19 and they are saying that they cannot get vaccinated because they do not believe that there is COVID-19”* [Female participant _ Urban].

*“You see they are telling us that there is COVID-19 but personally I never trusted that there is COVID-19 because I have not seen anyone suffering from COVID-19*, *just see the story of my blood brother*, *so I have never been motivated to accept this vaccine*,*”* [Female participant_ semi-urban].

One participant clearly indicated that they believed COVID-19 is just about business, and it has nothing to do with the disease. The participant further noted government officials were on board with the business people:

*“this disease is about big people’s business*, *not sure whether you understand me*? *So do you expect us to accept the vaccine*? *I am not sure who will agree to be vaccinated because myself I do not agree*. *This is people’s business*. *The government officials are collaborating with these other people on this business*. *The xxx (religious leader) is right that COVID-19 is about big people’s business”* [Female participant_semi urban].

Participants also indicated a range of questions (Table 3) on COVID-19 vaccines that they wanted to be answered by the government. Some questions reflect doubts and fear with regard to the COVID-19 vaccine. The dimensions of the questions related mainly to the eligibility criteria for COVID-19 vaccines, side effects of COVID-19 vaccines, quality of vaccines, the benefit of COVID-19 vaccine, modality of providing COVID-19 vaccines, and duration between receipt of the first vaccine and the second jab.

## Discussion

The findings of this study suggest that COVID-19 vaccine hesitancy persists among both rural and urban populations, despite the government’s effort to combat it.

Additionally, our study revealed comparable attitudes towards COVID-19 vaccination in both rural and urban areas of Tanzania, suggesting that vaccine hesitancy discourse has permeated widespread communities. The participants’ reluctance towards COVID-19 vaccines appears to be a reciprocal process influenced by broader factors that shape their perceptions of the benefits and risks of vaccination.

Vaccine related factors, such as concerns about vaccine safety, are significant determinant of vaccine hesitancy. This finding is of particular importance, as there is currently limited research exploring how individuals perceive COVID vaccine in East African region, including in Tanzania. Our vaccine-related findings are consistent with the international research, including a study conducted in Portugal, which identified low confidence in the COVID-19 vaccine and concerns over its safety and efficacy as key reasons for vaccine hesitancy [42].

Other studies have corroborated our findings on the impact of vaccine-related factors on vaccine hesitancy. A study conducted in China found that concerns over COVID-19 vaccine side-effects were among the most common reasons for vaccine hesitancy [43]. Similarly, a mixed method study in Nigeria revealed that risk perceptions and concerns about vaccine safety were barriers to COVID-19 vaccine acceptance [44]. A content analysis of questions raised by participants of another study conducted in Portugal found that concerns and fears surrounding COVID-19 were commonly expressed [45], possibly influenced by reports of rare but serious adverse events associated with COVID-19 vaccination, such as vaccine-induced immune thrombocytopenia with thrombosis, cardiac arrest, and deaths [46–48].

It is important to note that the majority of adverse events associated with COVID-19 vaccination are mild and temporary, such as local pain at the injection site, headache, fever, and fatigue [49]. However, in our study some participants expressed existing beliefs that accepting the COVID-19 vaccine could result in infertility or zombification. These beliefs are not supported by scientific evidence. Tanzanian health officials have been facing significant challenges in dispelling misinformation about COVID-19 vaccines. These challenges have emerged amid efforts to contain a third wave of infections in the country [50].

Studies have shown that providing factually accurate information about the side effects of the COVID-19 vaccines is crucial for promoting vaccine uptake. In China, a proof of vaccine safety and assurance of a low risk of COVID-19 infection were identified as the two most persuasive factors in promoting COVID-19 vaccine acceptance [43]. Additionally, it is essential to establish an effective health surveillance system at both the community and national levels to capture all adverse effects following COVID-19 vaccination. This will help to ensure that adverse effects are promptly and appropriately managed and strengthen people’s trust on the current COVID-19 vaccination program. A study in India found that health systems unpreparedness in monitoring COVID-19 vaccine side effects resulted in confusion among the families and contributed to vaccine hesitancy in communities [46].

The findings also found that knowledge on COVID-19 and the vaccine is insufficient to counter myths and misinformation that contribute to vaccine hesitancy. Multiple questions from participants about the COVID-19 vaccines, as presented in Table 3, require careful clarification by authorities and relevant stakeholders. Failure to address these questions can perpetuate mistrust in the government’s recommendations on COVID-19 vaccines. One of the questions, specifically linked to vaccine hesitancy, was whether having chronic medical conditions such as diabetes, high blood pressure, HIV or being on chronic medication makes one ineligible for COVID-19 vaccination. Therefore, it is essential to address these concerns to dispel the myths and provide accurate information. Similarly, a previous study conducted in Tanzania found that stakeholders had multiple questions regarding the malaria vaccine, and knowledge on the vaccine was considered important [51] and was found to be a determinant of immunization uptake in the country [52].

One **political factor** that contributed to vaccine hesitancy was reported to be inconsistent messages on COVID-19 being provided by government leaders. For instance participants referred to early 2020 when the use of traditional and home remedies, such as steam inhalation, were included among the interventions promoted by the government to manage COVID-19 [16]. The severity of a disease is not always enough to motivate people to accept health recommendations, particularly when communities have lost trust in the government’s messages. In Portugal, inconsistent and contradictory information on the COVID-19 vaccine was identified as one of the factors contributing to the vaccine hesitancy [42]. Similarly, a review of COVID-19 vaccine hesitancy in Africa also found that misinformation and politicization of the COVID-19 pandemic can contribute to public hesitancy or mistrust [30]. Therefore, it is crucial to provide clear, accurate, and consistent information to the public to address concerns and build trust in health recommendations.

The influence of politics on vaccine acceptance is an important consideration in public health interventions. Another political factor that contributed to vaccine hesitancy is the belief that COVID-19 is not a real disease. This belief is perpetuated by inconsistent messages from government officials, as noted by the study participants. Despite evidence of COVID-19 morbidity and mortality in Tanzania, there is a persistent perception that COVID-19 is a western business strategy and that COVID is not ‘real’. Similar perceptions have been reported, participants in Nigeria had doubts about the existence of the pandemic and believed it was intentionally created [29]. These findings underscore the need for a supportive political environment that provides reliable and accessible evidence-based information to the public. This is critical not only for vaccine acceptance but also for the success of all interventions against the pandemic. Building trust among citizens is essential and may require dynamic and open interactions between public health authorities and the public, including two-way dialogue and communication and not just one way messaging [53]. Failure to address political factors contributing to vaccine hesitancy may undermine the efforts to control the pandemic.

Clear recommendations have been provided in the African region on the need for concerned stakeholders to help build public trust toward COVID-19 vaccines by disseminating simplified, yet valid, information about vaccination, debunking popular myths with facts and remaining impartial toward political and financial interests [30]. This can be supported by the observation that when Her Excellence President Samia Suluhu Hassan in Tanzania publicly had the Johnson & Johnson vaccination during a campaign kick-off in July 2021, there was an increased trend in vaccination uptake in the general public. Such political examples need to be sustained to support current and future vaccine uptake.

Another important finding was related to **social factors**, specifically the significant influence of peers on COVID-19 vaccine uptake. This finding lends support to the theory of planned behaviour (TPB) which partly suggests that close people in the society may influence individual intentions to accept a particular intervention [54]. This dimension also supports evidence that individual behaviour can be socially constructed and not solely a product of personal opinions [55]. People may construct their hesitancy towards COVID-19 vaccines based on the discourse of others. Bandura’s social cognitive theory also suggests that through observational learning, individuals are more likely to perform a desired behaviour if they observe others adopting that behaviour [56]. Social influence has been found to be a common determinant of health intervention acceptance in various parts of Africa, including in Tanzania [39, 51].

Finally, the study highlights multiple questions on COVID-19 vaccines that require careful clarification by authority and relevant stakeholders. This is similar to a content analysis of 293 questions submitted to online, radio, newspaper and TV channel forums during the first month of the pandemic in Portugal which found that about 230 participant’s questions contained doubts on vaccines [45]. To the best of our knowledge this is the first study in East Africa to document the country specific questions that people have on COVID-19 vaccines.

### Strengths and limitations

This study presents novel findings on perceptions of COVID-19 vaccines among a sample of individuals in Tanzania. Using a qualitative approach, this study provides in-depth insights into the underlying reasons for vaccine hesitancy, which can be used to inform targeted vaccination programs. Nevertheless, certain limitations of this study should be taken into account when interpreting its results. For instance, the small sample size and limited geographic scope may restrict the generalizability of the findings to the broader Tanzanian population. However, the study still offers valuable insights that could aid in crafting effective health messaging and promoting vaccine uptake in the country. Additionally, given that this study was conducted in 2021, there may be changes in public perceptions of COVID-19 vaccines as new information and government actions emerge. Therefore, it may be necessary to conduct further research to monitor changes in public opinion over time.

## Conclusion

This study found multiple social, political, and vaccine related factors that contribute to vaccine hesitancy in semi-urban and urban areas in Tanzania. These factors include limited knowledge of COVID-19 vaccines and their safety, misinformation on the COVID-19 pandemic, inconsistent messages from the government officials on the COVID-19 vaccine, and people’s preference for traditional and home remedies over vaccination. Tanzania is yet to achieve the COVID-19 vaccination target, therefore, the results from this study could be used to design specific interventions at the various levels identified to improve vaccine uptake. A consistent education approach that emphasizes the reality of COVID-19, dispels misinformation regarding vaccine safety, and provides timely responses to participants’ inquiries, while concurrently addressing political mistrust, may enhance people’s confidence in both vaccines and governmental institutions. The provision of additional information regarding COVID-19 and its associated risks, as well as evidence-based data on vaccine safety, is likely to mitigate concerns and uncertainties surrounding vaccination, particularly among individuals with comorbidities. Establishing trust between citizens and political leaders is an essential goal, achievable through effective two-way communication and dialogue. Collaborating with influential figures and networks at various levels, including religious leaders, local organizations, and community health workers, can facilitate this process.
